# Public stigma profile toward mental disorders across different university degrees in the University of Valencia (Spain)

**DOI:** 10.3389/fpsyt.2022.951894

**Published:** 2022-08-12

**Authors:** Juan C. Ruiz, Inmaculada Fuentes-Durá, Marta López-Gilberte, Carmen Dasí, Cristina Pardo-García, María C. Fuentes-Durán, Francisco Pérez-González, Ladislao Salmeron, Pau Soldevila-Matías, Joan Vila-Francés, Vicent Balanza-Martínez

**Affiliations:** ^1^Faculty of Psychology, University of Valencia, Valencia, Spain; ^2^Center for Biomedical Research in Mental Health Network (CIBERSAM), Instituto de Salud Carlos III (ISCIII), Madrid, Spain; ^3^INCLIVA Biomedical Research Institute, Valencia, Spain; ^4^Department of Medicine, University of Valencia, Valencia, Spain; ^5^Department of Applied Economics, University of Valencia, Valencia, Spain; ^6^Intelligent Data Analysis Laboratory (IDAL), University of Valencia, Valencia, Spain

**Keywords:** stigma, mental disorders, university students, attitudes, attributions, prejudice, stereotyping

## Abstract

**Background:**

A large proportion of studies carried out in recent years in different populations have shown that stigma toward mental disorders is highly prevalent. In the present study we conducted a comprehensive assessment of stigma to describe and compare stigma toward mental disorders in students enrolled in five different university degrees.

**Methods:**

Three hundred and twenty-five students from the University of Valencia (Spain), attending the second term of their first-degree courses in the faculties of medicine, psychology, teaching, economics, and data science participated in this cross-sectional study. Stigma was measured using: the Reported and Intended Behavior Scale (RIBS), the Scale of Community Attitudes toward Mental Illness (CAMI), the Attribution Questionnaire (AQ-27), and the Knowledge about Mental Illness test (KMI).

**Results:**

We found different patterns of stigma according to gender, the fact of knowing or living with a person with mental disorders and the university degree studied. Overall, women show fewer stigmatizing attitudes than men but similar stereotypes and prejudice toward people with mental disorders. However, the pattern of results across degrees is more complex. Overall, students of medicine, psychology and teaching showed fewer stigmatizing attitudes than students of economics and data science but differences between degrees were more subtle in stereotypes and prejudice toward people with mental disorders.

**Conclusion:**

Our study suggests the existence of different profiles of stigma in relation to mental disorders in university students. These profiles varied in relation with the degree being studied, gender and already knowing or living with a person with mental disorders.

## Introduction

Stigma is identified as one of the key issues in mental illness (MI) (1, 2). Stigmatizing stereotypes and prejudices toward MI cause discrimination and exclusion behaviors that increase self-stigma, delay seeking treatment and hinder social functioning in people with MI (3, 4).

Several models have been put forward attempting to understand stigma and to describe the components of this construct and their interrelations (5–9). These models largely agree that stigma is a complex and multidimensional construct encompassing several factors. The social cognition model (10) established that MI stigma encompasses three components: beliefs, attitudes, and behaviors. Firstly, erroneous or false social beliefs constitute stereotypes: general beliefs about the features, attributes, and behaviors. For instance, thinking that the mentally ill are dangerous, incompetent and responsible, e.g., that they are to blame for their MI (10). Secondly, prejudices are generalized attitudes toward members of a social group and involves emotional aspects (10). For example, feeling scared, angry, or benevolent toward individuals with a MI. Thirdly, discrimination is a behavior directed against a social group based on prejudice, in other words, the behavioral result of prejudice. For instance, an employer who does not hire a job applicant purely because of their having a MI. Discriminative behaviors also include a higher desire for social distance from those with MI.

Different studies have consistently found that university students show high levels of public stigma. Assessment of stigma in students is important because they can become the target audience for anti-stigma programmes (11, 12). A recent review concluded that the presence of stigma toward MI among medical university students is widespread, with a prevalence of up to 97% (13). Stigmatizing attitudes and desire for more social distance have also been found among psychology students (14, 15). These studies also found that being familiar with individuals with psychiatric disorders or having had to visit a psychologist for personal reasons were factors associated with less social distance from people with MI. When it comes to the management of people with poor mental health, such findings for future health care professionals may result in negative consequences. A study in New Zealand found that most psychology students had stereotypes such as beliefs that mental patients are unpredictable, antisocial, and dangerous (16). Similar stigma-related issues have also been found among students of other health science degrees, such as nursing and pharmacy (17, 18). Fewer studies have been conducted in degrees that were non-healthcare related.

Despite growing research in this area, very few studies have compared this topic across different university degrees. Moreover, most of these comparative studies only assessed specific aspects of stigma. For instance, medical and dental university students showed more willingness to interact with a person labeled as mentally ill, e.g., less negative attitudes, compared to social science and engineering undergraduates from Hong Kong (19). In a study focusing only on male undergraduates in the US, those in science, technology, engineering, and mathematics (STEM majors) reported lower mental health literacy (knowledge) as well as less positive attitudes and intention to seek help for mental health issues, compared to students in non-STEM majors (20). Moreover, students of social sciences, assessed with the Opening Minds Scale for Healthcare Providers (OMS-HC), had significantly lower explicit stigmatizing attitudes than engineering students from Canada (21). In addition, lower explicit stigmatizing attitudes were found in female students, in those with a history of MI, and in those who have had a close relationship with a person with a MI (21). In the US, psychology, counseling, and social work students had a similar need for social distance from people with MI, as measured by the Social Distance Scale (22).

The results of other studies challenge the notion that stigmatizing behaviors toward the mentally ill are less severe in non-Western societies. Compared to medical students, arts/humanities and science/technology students from Nigeria showed a higher desire for social distance toward people with MI, in a modified version of Bogardus Social Distance Scale (23). In addition, female gender and not having a relative with a MI were predictors of high desire for social distance (23). In another study conducted in Nigeria, pharmacy students had more positive attitudes toward MI than those from teaching, arts, and social science colleges (24). Nevertheless, male gender, older age, a previous visit to a mental hospital and having a relative or friend with a MI, all significantly contributed to having fewer stigmatizing attitudes. Other studies compared stereotypes across degree courses in non-Western universities, which is relevant given that stereotypes are culturally defined (10). In Egypt, students enrolled in science degrees had more positive beliefs toward MI, assessed with the Belief about Mental Illness Scale, compared to medicine and pharmacy students (17). Specifically, pharmacy students self-reported that mentally ill people are dangerous and that mental illnesses take more time to heal than physical illnesses. Moreover, Qatari students showed significant rates of negative knowledge, attitudes, and beliefs about MI (25). Overall, these outcomes were more favorable among students enrolled in non-science-based colleges (comprising law, business, teaching, arts, and Islamic studies) compared to those in science-based degrees (comprising medicine, pharmacy, engineering and general sciences) (25).

In all, very few studies so far have compared stigma-related outcomes across different university degree courses nor have they employed a comprehensive assessment of the different aspects of stigma toward MI. The present study was designed to bridge that research gap. We adopted a multidimensional perspective to describe and compare stigma toward mental health among students enrolled in five different university degrees: Teaching, Economics, Data Science, Psychology and Medicine. The rationale to choose these degrees is as follows. Future teachers are the ones who will educate children and adolescents about this topic and this could therefore help them in the early detection of MI, which is a key factor in prognosis. In addition, future teachers can transmit their attitudes and behaviors to students, which will in turn have an impact on society. We were also interested in recruiting students enrolled in other degrees which have received far less attention previously, such as economics and data science students. The former can be involved in hiring employees in the future, so establishing the degree of MI stigma in this group seems key to approach the likelihood of integrating patients with mental health problems into the work market (26). Finally, data science students represent a group not involved in the future healthcare provision, education, and employment of individuals with a MI. Furthermore, we also aimed to analyse the attitudes toward MI of future clinicians and psychologists because they will be the future healthcare providers to people with MI.

In Spain, studies with university students have shown that medical and nursing students had more negative attitudes than psychology and occupational therapy students in several stigma-related themes: recovery, dangerousness, uncomfortability, disclosure, and discriminatory behavior (27). In another study evaluating the effect of internships in the last courses in the degrees of nursing, psychology and occupational therapy, results showed that although the effect was significant in the reduction of stigma toward people diagnosed with severe mental disorder in the degrees of nursing and psychology, it was small (28). Spanish teaching students have also participated in stigma studies. In a study comparing different countries with teaching students in different courses levels, results showed that the highest rates of stigma were in Spain and the lowest were in Canada, while Russia displayed intermediate values (29).

The aim of our study was to describe and compare stigma toward MI among students enrolled in the abovementioned five university degrees.

## Methods

### Participants

The questionnaires were administered to 325 undergraduate students from the University of Valencia (Spain) in their first year of the degrees of medicine (*n* = 69), psychology (*n* = 90), teaching (*n* = 70), economics (*n* = 46) and data science (*n* = 50). The participants' ages ranged from 18 to 54 years (M = 19.82; SD = 4.23), and 68.62% were females. A convenience sampling procedure was used. Consent was obtained from teachers in each degree to recruit participants during their class time. During class, students were asked to participate in the study and at that time they completed the questionnaires. In our sample, since the population size of students enrolled in first course of the target degrees was 1,545 people, and assuming a probability p = q = 0.50 and a confidence level of 95%, the sampling error was 4.8%. Sampling errors for each group were: 10.9% in teaching, 9.3% in psychology, 10.5 in medicine, 12.8 in economics and 6.7 in data science.

### Instruments

#### Knowledge about mental illness test

This test (30) measures knowledge about MI using 13 true, false, and not sure items that assess the level of knowledge that respondents have about MI, its causes, and possibilities of recovery. KMI score was computed as the number of correct responses, with higher scores indicating more knowledge about MI.

#### Reported and intended behavior scale

This scale (31) has eight items divided into two groups that measure familiarity, contact, and intentions to have contact with people with MI. The first four items ask the respondent about their familiarity and contact with people with MI using yes or no responses. We used these four items to identify and to assess the percentage of students that have known or know someone with a MI and the percentage of students that have lived/worked or are living/working with a person with MI.

#### Scale of community attitudes toward mental illness

This scale (32) evaluates the attitudes of the general population toward people with MI, focusing on opinions regarding the integration of people with MI in the community. It has 40 items with a five-point Likert scale format grouped in four dimensions (authoritarianism: the belief that people with MI are inferior and must be treated coercively; benevolence: a sympathetic view for those experiencing MI based on humanistic parameters; social restrictiveness: a view that the mentally ill are a threat to society; and community mental health ideology: concerned with the therapeutic value of the community and acceptance of de-institutionalized care). The Spanish version of the scale had a Cronbach α of 0.86 (33).

#### Attribution questionnaire (AQ-27)

This questionnaire was developed by Corrigan et al. (34) and measures stigma toward people with MI. It describes briefly a man diagnosed with schizophrenia that lives alone, works as a lawyer and has been hospitalized several times because of his illness. The 27 items of the questionnaire evaluate stereotypes using a 9-point Likert scale. The 27 items are grouped in 9 factors (responsibility, pity, anger, dangerousness, fear, help, coercion, segregation, and avoidance). Higher scores indicate higher values in that factor. The Spanish version of the questionnaire (35) had a Cronbach α of 0.86

### Procedure

All the participants completed the questionnaires through an online survey using the survey application tool LimeSurvey (https://www.limesurvey.org/es/) between February and March during the second term of the first-year degree course. Prior to their participation, the participants gave their written informed consent. The study was approved by the Ethics Committee of the University of Valencia.

### Statistical analysis

All statistical analyses were performed using the IBM-SPSS v.26 statistical package. Summary statistics were carried out through frequencies and percentages for categorical variables and by means and standard deviations for quantitative variables. Initially, the distributions of categorical variables were compared through Pearson's Chi-Squared tests, and the differences in age and KMI as a function of degree with ANOVAs. Then, different one-way between groups multivariate analysis of variance (MANOVA) were calculated to explore differences in the CAMI and AQ-27 tests in terms of gender, knowledge of people with MI, living with a person with MI and student group. *Post-hoc* analyses were computed using Tukey's multiple comparisons test to analyse differences between student groups.

## Results

A total of 325 students completed the full survey. Table 1 shows the distribution of students in the different undergraduate degrees in terms of gender, familiarity with mental health problems and results in the KMI test. In the groups of medicine, psychology, and teaching, the proportion of women over men was higher, but it was lower in the economics and data science groups. However, the distribution of students that know or have known a person with MI was the same in all groups, as was the case for students that live or have lived with a person with MI. The ANOVAs for age and KMI results revealed that both variables were significant. *Post-hoc* comparisons showed significant pairwise differences between medicine and economic students in terms of age and between several groups in terms of knowledge about MI as assessed by the KMI test (see Table 1).

**Table 1:** Demographic characteristics and scores in the KMI and RIBS in the five university degrees.

	**Medicine**	**Psychology**	**Teaching**	**Economics**	**Data science**	**Statistic**
	**(*n* = 69)**	**(*n* = 90)**	**(*n* = 70)**	**(*n* = 46)**	**(*n* = 50)**	
Age M (SD), years	20.70 (5.38)	20.24 (5.25)	20.00 (3.87)	18.37 (0.83)	18.94 (1.41)	*F* = 2.96, *p* = 0.02^a^
Gender, *n (%)*				
Men Women Other	15 (21.70) 54 (78.30) 0	12 (13.30) 77 (85.60) 1 (1.10)	15 (21.40) 55 (78.60) 0	28 (60.90) 17 (37.00) 1 (2.20)	32 (64.00) 17 (34.00) 1 (2.00)	*x*^2^ = 67.43, *p* <0.001
RIBS-Know, *n* (%)				
Yes No	53 (76.80) 16 (23.20)	73 (81.10) 17 (18.90)	49 (70.00) 21 (30.00)	31 (67.40) 15 (32.60)	32 (64.00) 18 (36.00)	*x*^2^ = 6.65, *p* = 0.156
RIBS-Live/work, *n* (%)				
Yes No	34 (49.30) 35 (50.70)	42 (46.70) 48 (53.30)	40 (57,10) 30 (42.90)	19 (41,30) 27 (58.70)	19 (38.00) 31 (62.00)	*x*^2^ = 5.24, *p* = 0.264
KMI M (SD)	10.51 (1.29)	10.97 (1.17)	9.76 (1.62)	10.26 (1.50)	10.68 (1.30)	*F* = 8.29, *p* <0.001^b^

a *Post hoc comparisons: Medicine > economics.*

b *Post hoc comparisons: Medicine > teacher; psychology > teacher and economics; data science > teacher.*

*RIBS: Familiarity with people with mental illness: Live/work with a person with mental illness; Know a person with a mental illness.*

*KMI: Knowledge of mental illness test*.

### Agreement with the statements in each AQ-27 items

Table 2 shows the results in the AQ-27 questionnaire expressed as the percentage of students that agree with the statement in each item of the questionnaire, scores 7 to 9 in the item (31). Only a small percentage of students think that people with MI are responsible for their illness (0.92–18.15%) and that they should be separated from their community (2.46–5.54%). The percentage of students who feel anger or fear toward persons with MI or perceive them as dangerous was also low (anger: 1.23–2.77%; fear: 3.08–4.31%; dangerousness: 1.23–6.77%). In line with these low stigmatizing attitudes, a high percentage of students are willing to help people with MI (69.54–85.54%) or will not avoid them (49.23–62.77%). Results also showed that although around fifty percent of students have feelings of pity or attitudes of concern toward people with MI (26–77%−59.38%) they are in favor of forcing patients to medicate or seek medical help (20.31–77.54%).

**Table 2:** Means and standard deviations of the scores for each item and percentage of participants that agree with the statement (percentage of participants that score 7, 8 or 9 in the item) in each AQ-27 item (*n* = 325).

	**Mean (SD)**	**%**
Responsibility^*^		
10. I would think that it is José's fault that he is in his current situation	1.30 (0.95)	0.92
11. To what extent do you believe that the cause of José's current situation is controllable?	4.42 (2.07)	18.15
23. In your opinion, to what degree is José responsible for his current situation?	2.51 (1.81)	4.31
**Pity**		
9. I would feel pity for José	4.67 (2.42)	26.77
22. To what extent do you understand José?	6.58 (2.03)	59.38
27. How much concern would you feel for José?	6.10 (2.21)	50.15
Anger^*^		
1. I would feel violent because of José	3.04 (1.65)	2.77
4. To what extent would you feel angry with José?	1.56 (1.19)	1.23
12. To what extent would you feel irritated by José?	2.40 (1.60)	2.77
Dangerousness^*^		
2. I would feel unsafe around José	3.40 (1.84)	7.08
13. In your opinion, to what extent is José dangerous?	3.50 (1.70)	6.77
18. I would feel threatened by José	2.28 (1.49)	1.23
Fear^*^		
3. José would terrify me	2.38 (1.59)	3.38
19. To what extent would José scare you?	2.73 (1.68)	4.31
24. To what extent would José terrify you?	2.47 (1.67)	3.08
**Help** ^**∧**^		
8. I would be willing to talk to José about his problem	7.95 (1.5)	85.54
20. What is the probability that you would help José?	7.72 (1.50)	80.31
21. With what certainty do you think you would help José?	7.23 (1.74)	69.54
Coercion^*^		
5. If I were in charge of José's treatment, I would require him to take his medication	7.47 (1.97)	77.54
14. To what extent do you agree that José should be forced to seek medical treatment even if he does not want to?	6.21 (2.24)	51.08
25. If I were in charge of José's treatment, I would force him to live in a supervised apartment	4.26 (2.25)	20.31
Segregation^*^		
6. I think that José poses a risk to his neighbors if he is not hospitalized	2.93 (1.82)	5.54
15. I think it would be better for José's community if he was confined in a psychiatric hospital	2.36 (1.76)	4.00
17. To what extent do you think that a psychiatric hospital, where José could stay away from his neighbors, is the best place for him?	2.54 (1.69)	2.46
**Avoidance** ^**∧**^		
7. If I were an employer, I would interview José for a job	6.18 (2.16)	51.69
16. I would share a car pool with José every day	6.00 (2.26)	49.23
26. If I were a landlord, I would probably rent an apartment to José	6.62 (2.14)	62.77

* *High scores correspond to high scores in stigma.*

*^∧^High scores correspond to low scores in stigma*.

### Gender differences

Scores on all four CAMI subscales significantly differed between women and men. Overall, women have less authoritarian and socially restrictive attitudes than men, and more benevolent and accepting attitudes when it comes to integrating patients' rehabilitation within the community (Table 3). However, in the AQ-27, women and men only differed in one of the nine dimensions. Specifically, women were more willing to offer help to persons with MI than men (Table 4).

**Table 3:** Means and standard deviations in the CAMI scale dimensions.

	**Authoritarianism**	**Benevolence**	**Social restrictiveness**	**CMHI^a^**
Total score	21.94 (4.10)	42.97 (4.44)	17.97 (3.73)	40.78 (5.14)
**Gender**				
Women (*n* = 220) Men (*n* = 102)	21.34 (3.98) 23.16 (4.10) F = 14.24; *p* <0.001	43.94 (3.83) 41.03 (4.90) F = 33.51; *p* <0.001	17.58 (3.52) 18.75 (4.03) F = 7.04; *p* = 0.008	40.72 (4.92) 39.59 (5.44) F = 8.67; *p* = 0.003
**RIBS-Know**				
Yes (*n* = 238) No (*n* = 87)	21.75 (4.10) 22.48 (4.07) F = 2.05; *p* = 0.153	43.09 (4.39) 42.63 (4.59) F = 0.67; *p* = 0.413	17.89 (3.70) 18.18 (3.84) F = 0.39; *p* = 0.532	41.00 (5.19) 40.15 (4.97) F = 1.77; *p* = 0.185
**RIBS-Live/work**				
Yes (*n* = 154) No (*n* = 171)	21.14 (4.07) 22.67 (4.00) F = 11.75; *p* <0.001	43.74 (4.12) 42.27 (4.61) F = 9.12; *p* = 0.003	17.48 (3.81) 18.41 (3.62) F = 5.08; *p* = 0.025	41.53 (4.91) 40.09 (5.26) F = 6.46; *p* = 0.012
**Degree** ^b^				
Medicine Psychology Teaching Economics Data science	20.86 (3.71) 20.73 (3.60) 22.56 (4.34) 24.48 (4.00) 22.44 (4.00) F = 8.95; *p* <0.001	43.83 (4.69) 44.58 (3.19) 43.40 (3.64) 39.76 (4.50) 41.22 (5.02) F = 13.51; *p* <0.001	17.97 (3.66) 16.76 (3.05) 17.87 (3.67) 20.26 (4.31) 18.18 (3.59) F = 7.29; *p* <0.001	41.32 (5.20) 42.54 (4.35) 40.93 (4.31) 37.59 (6.03) 39.56 (5.10) F = 8.77; *p* <0.001
	Medicine < Economics Psychology < Teaching Psychology < Economics	Medicine > Economics Medicine > Data science Psychology > Economics Psychology > Data science Teaching > Economics Teaching > Data science	Medicine < Economics Psychology < Economics Teaching < Economics Data science < Economics	Medicine > Economics Psychology > Economics Psychology > Data science Teaching > Economics

a *CMHI: Community mental health ideology;*

b *Post hoc comparisons between degrees.*

*F: Results of the ANOVAs comparing gender, familiarity and contact with mental illness, and degree*.

**Table 4:** Means and standard deviations in the AQ-27 dimensions.

	Responsibility^*^	Pity^*^	Anger^*^	Dangerousness^*^	Fear^*^	**Help^∧^**	Coercion^*^	Segregation^*^	**Avoidance^∧^**
Total score	8.22 (3.50)	17.35 (4.36)	7.00 (3.41)	9.19 (4.38)	7.59 (4.53)	22.90 (3.96)	17.93 (4.94)	7.82 (4.49)	11.21 (4.98)
**Gender**									
Women (*n* = 220) Men (*n* = 102)	8.26 (3.58) 8.05 (3.31) F = 0.25; *p* = 0.616	17.38 (4.07) 17.45 (4.89) F = 0.02; *p* = 0.887	6.79 (3.43) 7.46 (3.39) F = 2.68; *p* = 0.103	9.06 (4.19) 9.53 (4.80) F = 0.78; *p* = 0.377	7.55 (4.43) 7.72 (4.78) F = 0.09; *p* = 0.768	23.49 (3.76) 21.78 (4.00) F = 13.77; *p* <0.001	18.05 (4.90) 17.75 (5.05) F = 0.26; *p* = 0.613	7.59 (4.45) 8.35 (4.59) F = 2.00; *p* = 0.158	10.84 (5.02) 11.96 (4.84) F = 3.55; *p* = 0.060
**RIBS-Know**									
Yes (*n* = 287) No (*n* = 38)	8.34 (3.48) 7.92 (3.56) F = 0.90; *p* = 0.343	17.39 (4.34) 17.25 (4.42) F = 0.06; *p* = 0.801	6.98 (3.57) 7.06 (2.95) F = 0.03; *p* = 0.862	9.19 (4.50) 9.20 (4.05) F = 0.01; *p* = 0.991	7.63 (4.60) 7.48 (4.03) F = 0.07; *p* = 0.795	22.96 (4.00) 22.74 (3.87) F = 0.21; *p* = 0.649	17.96 (4.94) 17.86 (4.98) F = 0.03; *p* = 0.872	7.86 (4.59) 7.71 (4.24) F = 0.07; *p* = 0.798	10.98 (4.91) 11.84 (5.16) F = 1.90; *p* = 0.169
**RIBS-Live/work**									
Yes (*n* = 154) No (*n* = 171)	8.10 (3.65) 8.34 (3.36) F = 0.39; *p* = 0.535	17.05 (4.05) 17.63 (4.61) F = 1.41; *p* = 0.237	6.71 (3.43) 7.27 (3.38) F = 2.20; *p* = 0.139	8.77 (4.20) 9.57 (4.51) F = 2.69; *p* = 0.102	6.95 (3.85) 8.16 (5.00) F = 5.87; *p* = 0.016	23.38 (3.78) 22.47 (4.08) F = 4.37; *p* = 0.037	17.62 (4.94) 18.22 (4.94) F = 1.17; *p* = 0.281	7.12 (4.25) 8.45 (4.62) F = 7.27; *p* = 0.007	10.54 (4.63) 11.81 (5.22) F = 5.37; *p* = 0.021
**Degree** ^**a**^									
Medicine Psychology Teaching Economics Data science	7.71 (3.69) 8.91 (2.97) 7.80 (3.93) 8.41 (3.309 8.12 (3.58) F = 1.55; *p* = 0.187	16.62 (4.38) 17.47 (3.76) 18.17 (4.44) 17.70 (5.06) 16.70 (4.45) F = 1.48; *p* = 0.209	5.99 (2.77) 6.49 (3.04) 7.86 (3.90) 8.35 (3.57) 6.90 (3.41) F = 5.20; *p* <0.001	8.12 (4.29) 8.89 (3.82) 9.54 (4.35) 10.74 (5.01) 9.30 (4.54) F = 2.77; *p* = 0.028	6.51 (3.74) 7.08 (4.06) 8.34 (4.67) 8.59 (4.96) 8.04 (5.37) F = 2.48; *p* = 0.044	23.97 (3.34) 23.60 (3.59) 23.03 (3.96) 21.59 (3.77) 21.20 (4.76) F = 5.88; *p* <0.001	17.77 (4.48) 18.13 (4.42) 16-91 (5.80) 18.98 (4.93) 18.28 (5.07) F = 1.38; *p* = 0.240	6.67 (3.50) 7.54 (4.00) 7.79 (5.06) 9.93 (5.52) 8.00 (4.14) F = 3.93; *p* = 0.004	9.87 (4.72) 11.08 (5.02) 11.57 (4.95) 12.87 (4.78) 11.26 (5.16) F = 2.69; *p* = 0.031
			Medicine < Teaching Medicine < Economics Psychology < Economics	Medicine < Economics		Medicine > Economics Medicine > Data science Psychology > Economics Psychology >‘Data science		Medicine < Economics Psychology < Economics	Medicine < Economics

* *High scores correspond to high scores in stigma;*

*^∧^High scores correspond to low scores in stigma;*

*^a^Post hoc comparison between degrees.*

*F: Results of the ANOVAs comparing gender, familiarity and contact with mental illness, and degree*.

### Differences as a function of contact with people with mental illness

There were no significant differences in stigma in the students when considering whether they knew or have known people with MI in any of the subscales of the CAMI nor in any of the AQ-27 dimensions. However, the fact of living or working with or having lived or worked with a person with MI revealed significant differences in comparison with students that do/did not. Analysis of the CAMI subscales (Table 3) showed that students living/having lived or working/having worked with a person with MI have more benevolent and accepting attitudes related with the integration of patients' rehabilitation within the community and also greater willingness to help. At the same time, they have less authoritarian and restrictive attitudes. Those students also have lower negative attitudes of fear, segregation and avoidance toward people with MI and higher helping attitudes, as assessed by the AQ-27 (Table 4).

### Differences between university degrees

Significant differences were found in the four subscales of the CAMI (Table 3) and in six subscales of the AQ-27 (Table 4) but there was not a homogenous pattern of differences between groups. Overall, in the CAMI, medicine, psychology and teaching students show more positive benevolent attitudes and accepting attitudes related with the integration of patients' rehabilitation within the community than students of economics and data science. Moreover, the former group showed less authoritarian and socially restrictive attitudes. However, differences between teaching and data science students were significant in benevolence attitudes only. Regarding the AQ-27, medical students showed more positive attitudes in anger, dangerousness, fear, help, segregation and avoidance than economic students. Psychology students also showed more positive attitudes than their peers in economics in terms of anger, help and segregation. Furthermore, medicine and psychology students showed more positive help attitudes than students of data science. Lastly, fewer anger attitudes were shown by students of medicine than those of teaching.

## Discussion

Stigma toward MI is a public health problem because of the impact it has on the lives of people with MI, creating barriers in employment opportunities, independent living, and recourse to health services.

Overall, the results of our study show that only a small percentage of students think that persons with MI are responsible for their illness and should be separated from their community, feel anger or fear or perceive them as dangerous and a high percentage of students are willing to help people with MI or do not intend to avoid them. Furthermore, it is evident that around half of all students have feelings of pity or attitudes of concern, and they are in favor of forcing patients to medicate or seek medical help.

Some relevant findings arose regarding beliefs, attitudes and behaviors toward people with MI, in terms of gender, knowledge of people with MI, living with a person with MI and student degree.

Firstly, we found that women have fewer authoritarian and socially restrictive attitudes than men, more benevolent and accepting attitudes related with the integration of patients' rehabilitation within the community and are more willing to offer help. These results are in line with previous studies (21, 23, 36, 37). Nonetheless, some studies have found opposite results, for example, a higher proportion of women than men stated that they would feel afraid to have a conversation with someone diagnosed with schizophrenia (38) or have not found differences (39). These results could be explained by two arguments that have been put forward in the literature: the general belief that men can manage their mental problems (40), and the idea that women behave differently from men in the face of MI, with women acting in a friendlier way (41, 42).

Secondly, there are no differences between degrees in the percentage of students knowing or living/working with a person with MI. In fact, the percentages in both cases were high for all degrees (between 64 and 81%; and between 38 and 57%, respectively). However only the fact of living/working is associated with lower stigma scores, specially in stigma behavior intentions. These results are in line with previous studies (19, 21, 23, 24) and support the idea of the relevance of incorporating people with MI as employees or in other daily life activities in the community for its probable effect in reducing stigma. They also support the strategy of including interpersonal contact with people with MI in anti-stigma interventions (10).

As members of a society, students cannot remain immune to societal influences characterized by the disrespect toward patients with MI (43). However, the nature of the chosen university programs probably already constitutes a different starting point in beliefs, attitudes and behaviors. In our study, medicine and psychology students showed less authoritarianism and social restriction and greater benevolence and CMHI. At the same time, economics showed the higher stigmatizing scores in this dimensions, and teaching and data science showed an intermediate score. There are also degree differences in beliefs (dangerousness), attitudes (anger), and behaviors (help, segregation and avoidance). Medicine and psychology students present the lowest stigmatizing scores and economics the highest. Medicine and psychology programs focus their attention on the care of health, including mental health This is also the objective of students when choosing to follow these degrees and could be the reason for their lower stigma scores.

Lack of knowledge, stereotypes and prejudices in mental health are usually common among students in secondary and university education, and this is why several authors point out the need to work with this population (44). Implementing anti-stigma strategies would have implications in reducing the different aspects that constitute social stigma and would benefit community integration. It is also important to remember that the WHO (1) points out that stigma is one of the most important problems related to mental health in contemporary society and mental illness-related stigma is present in every country (1, 45).

## Conclusions

Our study has delineated a stigma profile toward MI and has demonstrated the existence of stigma in university students and the existence of differences between the degrees. This justifies the need to introduce brief anti-stigma interventions taking into account the profile that characterizes each group. Research has shown that everyone can contribute to stigmatization (12) and that everyone has opportunities to fight against it (46), including institutions such as Universities that could launch anti-stigma intervention programs. Future studies should evaluate these programs.

Students from health sciences show more positive beliefs, attitudes and behaviors. However, these students will work with people with MI so interventions to reduce stigma among these students should be carried out continuously, because stigma is resilient and resistant to intervention (12).

In the case of teaching students, primary and secondary teachers often have a limited amount of mental health knowledge (47, 48) and do not feel confident about helping students with mental health problems (49). However, they can play an important role in the early identification of MI and in early intervention (50). It is therefore necessary that teaching students also participate in anti-stigma interventions, as is the case of economic students that, globally, have shown the highest levels of stigma.

### Limitations

This study has only analyzed five university degrees, it would be interesting to extend it to other degrees in science, health sciences and social sciences. In this work, validated and widely used evaluation instruments have been used in the field of stigma study (KMI, part of the RIBS, CAMI and AQ-27) (51), but many published studies use other instruments, which makes it difficult to make comparisons. To overcome this, it would be useful to reach expert consensus, as has been done in other fields (52) on the most appropriate instruments for the assessment of the different aspects involved in the stigmatization process.

Research based on self-reported data could favor information bias due to the social desirability effect. Another key issue in survey-based research is whether respondents differ from non-respondents in some way that is likely to impact systematically the prevalence of stigma issues. The use of convenience samples and self-reporting instruments are potential limitations for this study.

The present study also has some strengths. It is one of the few studies to compare stigma across several university degrees in Spain. Moreover, we employed a comprehensive assessment of the different aspects of stigma. In the Spanish population one out of ten people over the age of 15 (10.8%) suffers from some type of mental disorder and 2.1% of the population has some type of severe MI (53), which gives a good idea of the number of people who may be suffering the effects of stigmatizing beliefs, attitudes and behaviors. If, in addition, we take into account the fact that some studies have shown relatively high levels of stigma in the general population (54), the need to know the profile of stigma in the general population, and in subgroups within the population, such as university students, is justified in order to act accordingly to reduce public stigma. The aim of future studies should be to increase the sample of university students and incorporate other groups such as high school students, the general population, health and socio-health professionals, as well as professionals from other fields such as those related to world of work.

## Data availability statement

The raw data supporting the conclusions of this article will be made available by the authors, without unduereservation.

## Ethics statement

The studies involving human participants were reviewed and approved by Ethics Committee of Universidad of Valencia (Spain). The patients/participants provided their written informed consent to participate in this study.

## Author contributions

JCR, IF-D, VB-M, and ML-G contributed to the conception and design of this project and to the writing of the first draft of the manuscript. JCR, CD, PS-M, and JV-F conducted the methodology and analysis. CP-G, MF-D, FP-G, and LS acquired the data and made important contributions to sections of the manuscript. IF-D and CD obtained the funding. All authors contributed to the article and approved the submitted version.

## Funding

This study was supported by a grant from Special Actions of the University of Valencia (No. UV-INV-AE-1554362).

## Conflict of interest

The authors declare that the research was conducted in the absence of any commercial or financial relationships that could be construed as a potential conflict of interest.

## Publisher's note

All claims expressed in this article are solely those of the authors and do not necessarily represent those of their affiliated organizations, or those of the publisher, the editors and the reviewers. Any product that may be evaluated in this article, or claim that may be made by its manufacturer, is not guaranteed or endorsed by the publisher.
